# Supplementary material for: The interactome of intact mitochondria by cross-linking mass spectrometry provides evidence for coexisting respiratory supercomplexes
Source: Mol Cell Proteomics. 2017 Dec 8;17(2):216–32. doi: 10.1074/mcp.RA117.000470 (PMC5795388; doi:10.1074/mcp.RA117.000470)
Supplement: Supplemental Data [file supp_RA117.000470_133922_0_supp_23978_fzffwf.zip › spectra_annotation/mito_DR_spectra_annotation/372-1-1-1-19-1.pdf]

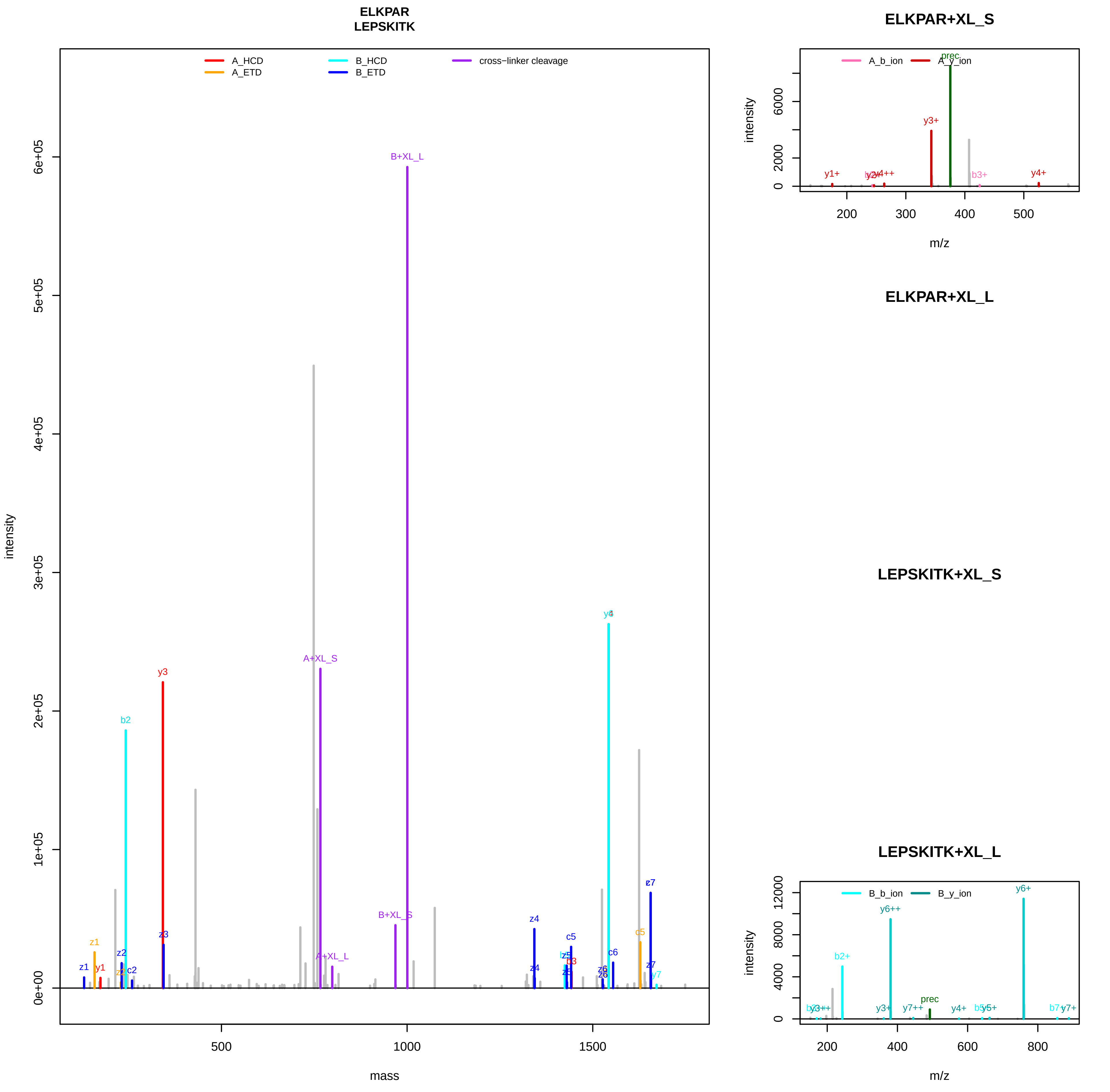

ELKPAR
LEPSKITK
A_HCD
A_ETD
B_HCD
B_ETD
cross−linker cleavage
B+XL_L
A+XL_S
B+XL_S
A+XL_L
y3
b2
z1
y1
z2
c2
z3
z4
c5
c6
z5
b3
z6
z7
y7
y4
c5
intensity
mass
0e+00
1e+05
2e+05
3e+05
4e+05
5e+05
6e+05
500
1000
1500
ELKPAR+XL_S
A_b_ion
A_y_ion
prec
y3+
y1+
y4+
b3+
intensity
m/z
0
2000
4000
6000
200
300
400
500
ELKPAR+XL_L
LEPSKITK+XL_S
LEPSKITK+XL_L
B_b_ion
B_y_ion
y6+
y6++
b2+
prec
y3+
y7++
y4+
y5+
b7+
y7+
intensity
m/z
0
4000
8000
12000
200
400
600
800
